# Selective Modulation of OTUB1 Noncanonical Function via a bioPhosTAC Strategy

**DOI:** 10.1002/advs.77265

**Published:** 2026-08-20

**Authors:** Seung Un Seo, Seon Min Woo, Minhyeong Choi, Soeun Rhee, Simmyung Yook, Donghyuk Shin, Kwang‐Su Park, Taeg Kyu Kwon

**Affiliations:** ^1^ Department of Immunology School of Medicine Keimyung University Daegu South Korea; ^2^ Department of Systems Biology College of Life Science and Biotechnology Yonsei University Seoul South Korea; ^3^ School of Pharmacy Sungkyunkwan University Suwon Gyeonggi South Korea; ^4^ College of Pharmacy Keimyung University Daegu South Korea; ^5^ Center for Forensic Pharmaceutical Science Keimyung University Daegu South Korea

**Keywords:** bioPhosTAC, OTUB1, phosphorylation, tyrosine phosphatase

## Abstract

OTUB1 is a canonical deubiquitinating enzyme that hydrolyzes K48‐linked polyubiquitin chains as well as a regulator of several noncanonical pathways controlled by site‐specific post‐translational modifications. Phosphorylation at tyrosine 26 (Y26) of OTUB1 recently emerged as a key regulatory switch that stabilizes Raptor (central component of mTORC1), thereby enhancing mTORC1 activity and influencing cell growth and metabolism. Selective modulation of Y26 phosphorylation offers an opportunity to elucidate the noncanonical roles of OTUB1 without perturbing its catalytic activity. However, genetic or kinase inhibition strategies lack the specificity to separate these functions. Here, a tyrosine‐specific targeted protein dephosphorylation strategy was developed by recruiting the protein tyrosine phosphatase, SHP2, to OTUB1 via a peptide‐based bifunctional molecule, termed bioPhosTAC. This bioPhosTAC comprises an OTUB1‐binding peptide linked to an SHP2‐binding peptide through a short linker, enabling proximity‐induced Y26 dephosphorylation. The bioPhosTAC selectively reduced OTUB1 Y26 phosphorylation resulted in Raptor‐related mitochondrial fusion and Bim upregulation. This study represents the first demonstration of a peptide‐based SHP2‐mediated bioPhosTAC system for selective tyrosine dephosphorylation through induced proximity. These findings provide a new chemical biology tool for dissecting the noncanonical roles of OTUB1 and broaden the scope of targeted protein dephosphorylation strategies to tyrosine phosphorylation, potentially allowing precise modulation of phospho‐dependent signaling.

## Graphical abstract

This work positions the versatile performance of the peptide‐based bioPhosTAC platform for dissecting phosphorylation‐dependent biology and expanding the scope of induced‐proximity technologies. We demonstrated that selective manipulation of a tyrosine phosphorylation site is sufficient to propagate coordinated cellular consequences. KOI‐pep‐mediated dephosphorylation of OTUB1 Y26 accelerated the proteasomal degradation of Raptor, disrupted mitochondrial dynamics, increased ROS generation, and ultimately triggered apoptosis.

## 1 Introduction

The deubiquitinase, OTUB1, has been extensively studied for its canonical role in hydrolyzing K48‐linked polyubiquitin chains, thereby regulating protein stability across diverse signaling pathways [1, 2]. The catalytic activity of OTUB1 is critical for maintaining protein homeostasis [3], immune responses [4], DNA damage repair [5], and the regulation of the cell cycle [6]. Beyond this enzymatic function, however, recent evidence has highlighted the noncanonical roles of OTUB1 [7, 8]. The α‐helix located in the N‐terminal of OTUB1 can interact with and inhibit the function of ubiquitin‐conjugated E2, thereby impeding ubiquitin transfer to substrate proteins [9]. These noncanonical functions are often modulated by post‐translational modifications, particularly site‐specific phosphorylation events in the N‐terminal of OTUB1. Phosphorylation at tyrosine 26 (Y26) of OTUB1 has emerged as a crucial regulatory switch for its noncanonical function, particularly in stabilizing Raptor, a core component of mechanistic target of rapamycin complex 1 (mTORC1) [10]. OTUB1‐mediated stabilization of Raptor enhances mTORC1 activity and contributes to the regulation of cell growth and metabolism [10]. This Y26‐dependent phosphorylation‐mediated axis provides a distinct regulatory mechanism that is separable from the deubiquitinating function of OTUB1. Accordingly, the selective modulation of Y26 phosphorylation presents a powerful strategy to dissect and control the noncanonical roles of OTUB1 without perturbing its canonical enzymatic activity. Conventional genetic strategies, such as knockdown/knockout of OTUB1, fail to differentiate between these two functional modes [7]. Similarly, inhibition of upstream tyrosine kinases, including SRC, which phosphorylates OTUB1 at Y26 [10], suffers from poor specificity because SRC also phosphorylates a wide range of other substrates [11]. Thus, a more selective and targeted approach is required to specifically modulate the phosphorylation state of OTUB1 at Y26.

Inspired by recent advances in induced proximity technologies, we envisioned a dephosphorylation strategy in which a phosphatase is selectively recruited to OTUB1 to promote Y26 dephosphorylation [12]. The phosphorylation targeting chimeras (PhosTAC) approach, which uses heterobifunctional molecules to induce serine or threonine dephosphorylation via PP2A recruitment, has demonstrated proof‐of‐concept for induced dephosphorylation [13, 14, 15]. However, this system has not been extended to regulate tyrosine phosphorylation, which requires distinct enzymatic mechanisms and biological contexts.

In this study, we developed a tool for selective tyrosine dephosphorylation by recruiting SHP2, a protein tyrosine phosphatase that is frequently overexpressed in cancer cells, exhibits broad substrate specificity, and plays diverse roles in oncogenic signaling pathways [16]. To achieve this, we designed a peptide‐based PhosTAC molecule, named bioPhosTAC, comprising one peptide that binds to OTUB1 and another that binds to SHP2. These two peptides are connected by a short linker sequence. This design enabled the selective dephosphorylation of phosphorylated OTUB1 at Y26 through a proximity‐driven mechanism. This new targeted protein dephosphorylation strategy enables the selective regulation of downstream signaling pathways mediated by phospho‐OTUB1 Y26. Consequently, the bioPhosTAC successfully led to mitochondria fusion that is downstream phenotype of phospho‐OTUB1 Y26/Raptor axis. Additionally, bioPhosTAC effectively induced apoptosis in triple‐negative breast cancer cell lines both in vitro and in vivo. Overall, this study presents a novel targeted protein dephosphorylation strategy and bioPhosTAC for phospho‐OTUB1 Y26, which could serve as a valuable peptide tool to further investigate phospho‐OTUB1 biochemistry. Notably, this is the first report of a peptide‐based, SHP2‐mediated bioPhosTAC system that selectively modulates tyrosine phosphorylation through induced proximity.

## 2 Results

### 2.1 Design and Validation of a Novel OTUB1 Binder

Despite the active interest in proximity‐based modalities, including chemically induced proximity drugs [17], OTUB1 itself has been poorly explored in terms of its binding ligands or peptides. To date, only a few OTUB1‐binding ligands have been described, including EN523, a covalent recruiter used in DUBTAC applications, and OTUB1/USP8‐IN‐1, a dual OTUB1–USP8 inhibitor [18, 19]. Although these molecules demonstrate that OTUB1 is chemically addressable, their covalent reactivity, dual‐target activity, and limited structural selectivity suggest potential constraints for broader induced‐proximity applications. Motivated by these considerations, we sought to identify a reversible, noncovalent peptide capable of selectively binding OTUB1. Using RFdiffusion, we generated a diverse set of de novo peptide candidates and performed initial triage based on predicted aligned error (pAE) at the peptide–OTUB1 interface to select high‐confidence binders. The shortlisted peptides were subjected to AlphaFold‐based complex prediction, and candidates with stable interface geometry and high pLDDT scores further prioritized. Through stepwise filtering pipeline, we ultimately identified a 19‐amino acid noncovalent peptide binder (Binder_19_) with the most robust predicted interaction with OTUB1 (Figure 1A). The designed peptide and OTUB1 complex were further evaluated with AlphaFold2. In the predicted model, Gln2 and Arg5 of Binder_19_ form hydrogen bonds with Thr131 and Gln184 of OTUB1, respectively. Additionally, Ile9 and Ile16 engage in hydrophobic contact with Thr134 and Tyr170, whereas Arg13 forms a salt bridge with Asp137. Together, these interactions support binding at a site distal from the catalytic residues of OTUB1 (Figure 1B).

**FIGURE 1:**
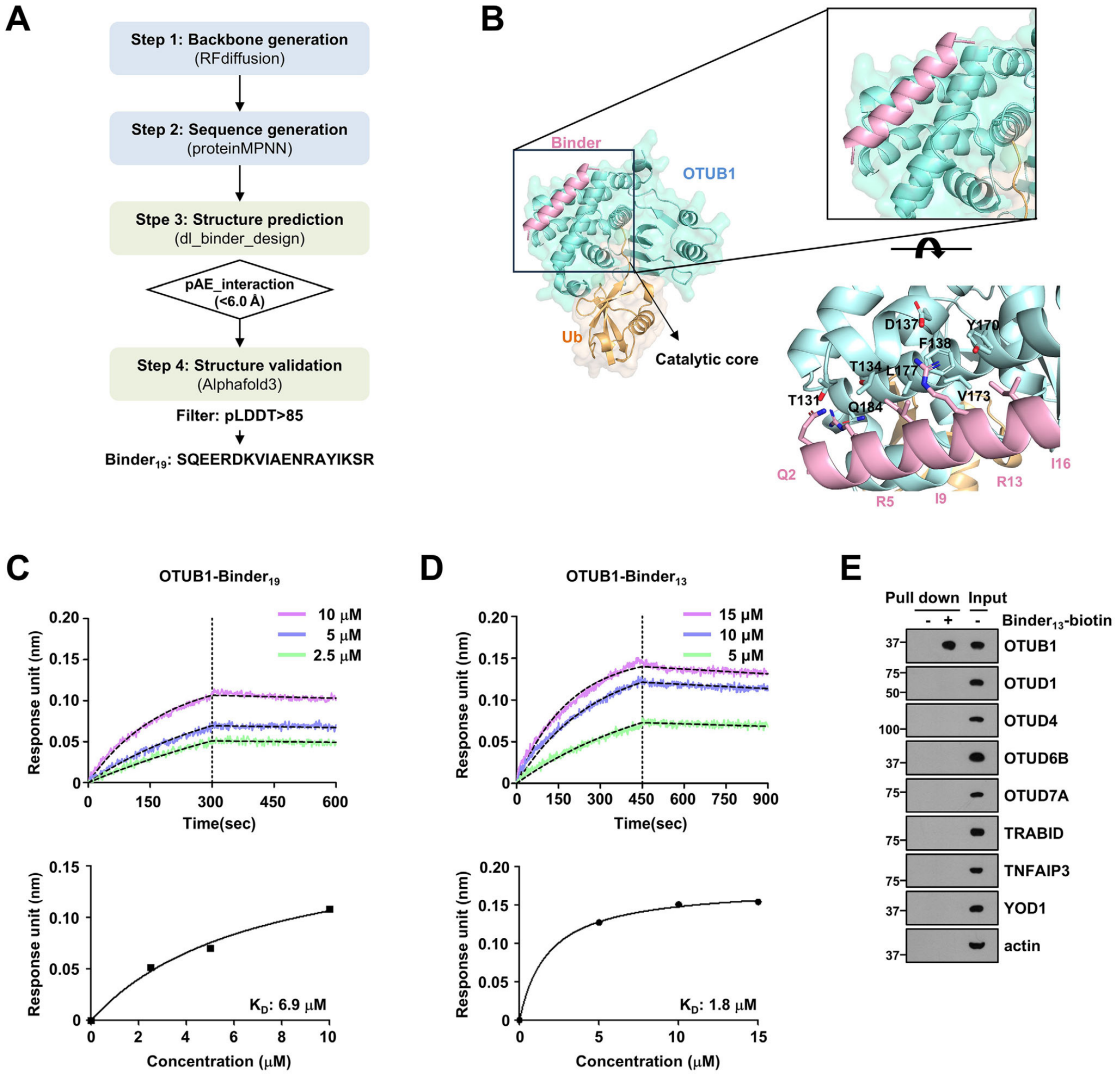
Design and validation of OTUB1 binders. (A) *De novo* binder design process using RFdiffusion for OTUB1. (B) Predicted complex structures of OTUB1_40–271_ (cyan), ubiquitin (orange), and Binder_19_ (pink) determined using AlphaFold2. Interacting residues are represented as stick models. The subset shows the catalytic site of OTUB1, and catalytic residues are shown as stick models. (C) Binding kinetics measurements of Binder_19_ to OTUB1_40–271_ were determined using BLI. One representative experiment is shown; similar results were obtained in independent experiments. (D) Binding kinetics measurements of Binder_13_ to OTUB1_40‐271_ were determined using BLI. One representative experiment is shown; similar results were obtained in independent experiments. (E) Pull‐down assay using Binder_13_‐biotin against several OTU family proteins.

Next, we validated the binding ability of Binder_19_ to OTUB1, which exhibited a dissociation constant (*K_d_*) toward OTUB1 within the micromolar range (Figure 1C). An additional consideration in designing a novel OTUB1 binder is the targeting of allosteric sites, because the catalytic core of OTUB1 is highly conserved among members of the OTU family. Therefore, Binder_19_ was designed to engage OTUB1 at a surface distinct from the catalytic core, functioning as an allosteric binder, consistent with the predicted binding pose (Figure 1B). As peptide binders designed for proximity‐based modalities require a reduced size to improve cell permeability and enable therapeutic application, we trimmed the initial 19‐mer peptide (SQEERDKVIAENRAYIKSR) to a shorter 13‐mer peptide (SQEERDKVIAENR; Binder_13_). Biolayer interferometry (BLI) assays confirmed that the truncation of Binder_19_ to Binder_13_ retained strong binding, indicating that the shorter peptide is sufficient for effective OTUB1 interaction (Figure 1D). To address the selectivity of Binder_13_, we performed pull‐down assays using biotin‐conjugated Binder_13_ against several OTU family deubiquitinases, including OTUD1, OTUD4, OTUD6B, OTUD7A, TRABID, TNFAIP3, and YOD1. The results demonstrated that Binder_13_ selectively binds to OTUB1, with negligible binding to the other tested OTU family proteins (Figure 1E).

### 2.2 Design of a bioPhosTAC for Phospho‐OTUB1

SHP2‐binding peptides have been previously reported [20], including the IRS1‐derived peptide, which is distinct from the one used for OTUB1. To design the peptide‐based bioPhosTAC targeting OTUB1 Y26 dephosphorylation, we constructed a peptide comprising an N‐terminal R7 cell‐penetrating sequence, followed by an IRS1‐derived motif for SHP2 binding, a 6‐aminohexanoic acid linker [21], and Binder_13_ at the C‐terminus to engage OTUB1. This construct is referred to as R7IO‐pep (Figure 2A).

**FIGURE 2:**
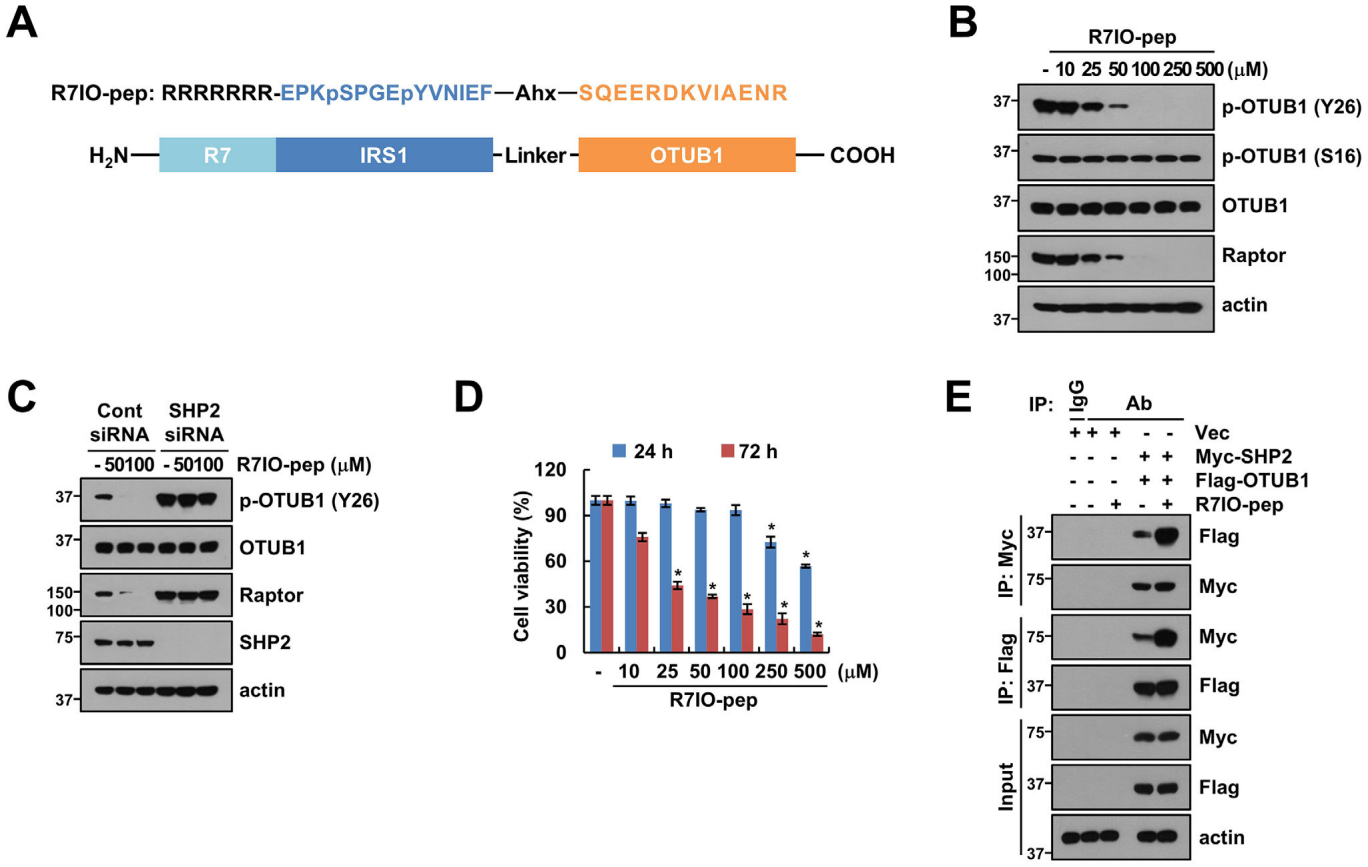
Design of a bioPhosTAC for phospho‐OTUB1. (A) Schematic view of the designed bioPhosTAC. (B) Western blotting of protein expression in MDA‐MB231 cells treated with 10–500 µM R7IO‐pep for 24 h. (C) Western blotting of protein expression in MDA‐MB231 cells transfected with control or SHP2 siRNA followed by 50–100 µM R7IO‐pep treatment for 24 h. (D) MTT assay for cell viability in MDA‐MB231 cells treated with 10–500 µM R7IO‐pep for 24–72 h. (E) Co‐immunoprecipitation analysis of the SHP2‐OTUB1 interaction. Lysates from Myc‐SHP2 and Flag‐OTUB1 co‐transfected MDA‐MB231 cells were incubated with by 100 µm R7IO‐pep treatment for 1 h. Values shown in the graphs represent the mean ± SD of results from three independent experiments. ^*^ *p* < 0.05 compared with the control.

To evaluate the performance of R7IO‐pep as a bioPhosTAC, we first assessed its ability to induce the selective dephosphorylation of OTUB1 at Y26. In a concentration‐dependent assay, R7IO‐pep selectively reduced phospho‐OTUB1 at Y26 while leaving phospho‐OTUB1 S16 unchanged, confirming its tyrosine phospho‐specific activity (Figure 2B). The predicted three‐dimensional structure of OTUB1 further illustrates that Y26 and S16 occupy distinct spatial positions (Figure S1A). As our previous work demonstrated that Raptor abundance is tightly coupled to the phosphorylation state of OTUB1 at Y26 rather than at S16 [10], we then examined downstream signaling. Consistent with its biochemical activity, R7IO‐pep led to a marked reduction in Raptor levels (Figure 2B). To test whether this effect was dependent on SHP2 recruitment, which is the key mechanistic driver of bioPhosTAC, we performed SHP2 knockdown experiments. Loss of SHP2 completely abolished both OTUB1 Y26 dephosphorylation and Raptor downregulation, confirming that R7IO‐pep activity requires SHP2 proximity (Figure 2C). Finally, we assessed whether these biochemical changes translated into antiproliferative effects in MDA‐MB231 cells, which rely on Raptor for survival [10, 22]. R7IO‐pep decreased the viability of MDA‐MB231 cells only at relatively high concentrations >100 µM. Consistent with these findings, prolonged exposure for 72 h further enhanced the antiproliferative effect of R7IO‐pep, supporting a time‐dependent cellular response (Figure 2D). To evaluate the ability of R7IO‐pep to induce proximity between the SHP2 and OTUB1, we performed an in vitro co‐immunoprecipitation assay. R7IO‐pep potentiated the interaction between Myc‐SHP2 and Flag‐OTUB1 (Figure 2E). These results support that R7IO‐pep promotes the interaction between SHP2 and OTUB1, consistent with ternary complex formation. Collectively, these results validate the mechanistic specificity of R7IO‐pep as a SHP2‐dependent bioPhosTAC capable of modulating OTUB1–Raptor signaling. However, because dephosphorylation and downstream effects were observed only at concentrations ≥50 µM, further structural optimization was required to improve system potency.

### 2.3 KKLC‐1, Tumor‐Homing Peptide, Enhances the Effect of bioPhosTAC

Initially, we hypothesized that the limited biological activity of the first‐generation bioPhosTAC was partly due to insufficient cellular uptake. To improve intracellular delivery, we replaced the R7 cell‐penetrating sequence with a peptide targeting Kita‐Kyushu lung cancer antigen 1 (KKLC‐1; CKNTALTTC), a cancer–testis antigen selectively expressed in malignant cells [23, 24, 25]. In refining the construct design, we also introduced phosphomimetic substitutions within the IRS1 module (pS→D and pY→E) to maintain a stable negative charge, given that native phospho‐residues may be susceptible to dephosphorylation or enzymatic cleavage inside cells. In addition, because the spatial arrangement of the IRS1‐ and OTUB1‐binding elements could influence proximity formation, we designed two peptide configurations in which the binding positions were reversed (Figure 3A).

**FIGURE 3:**
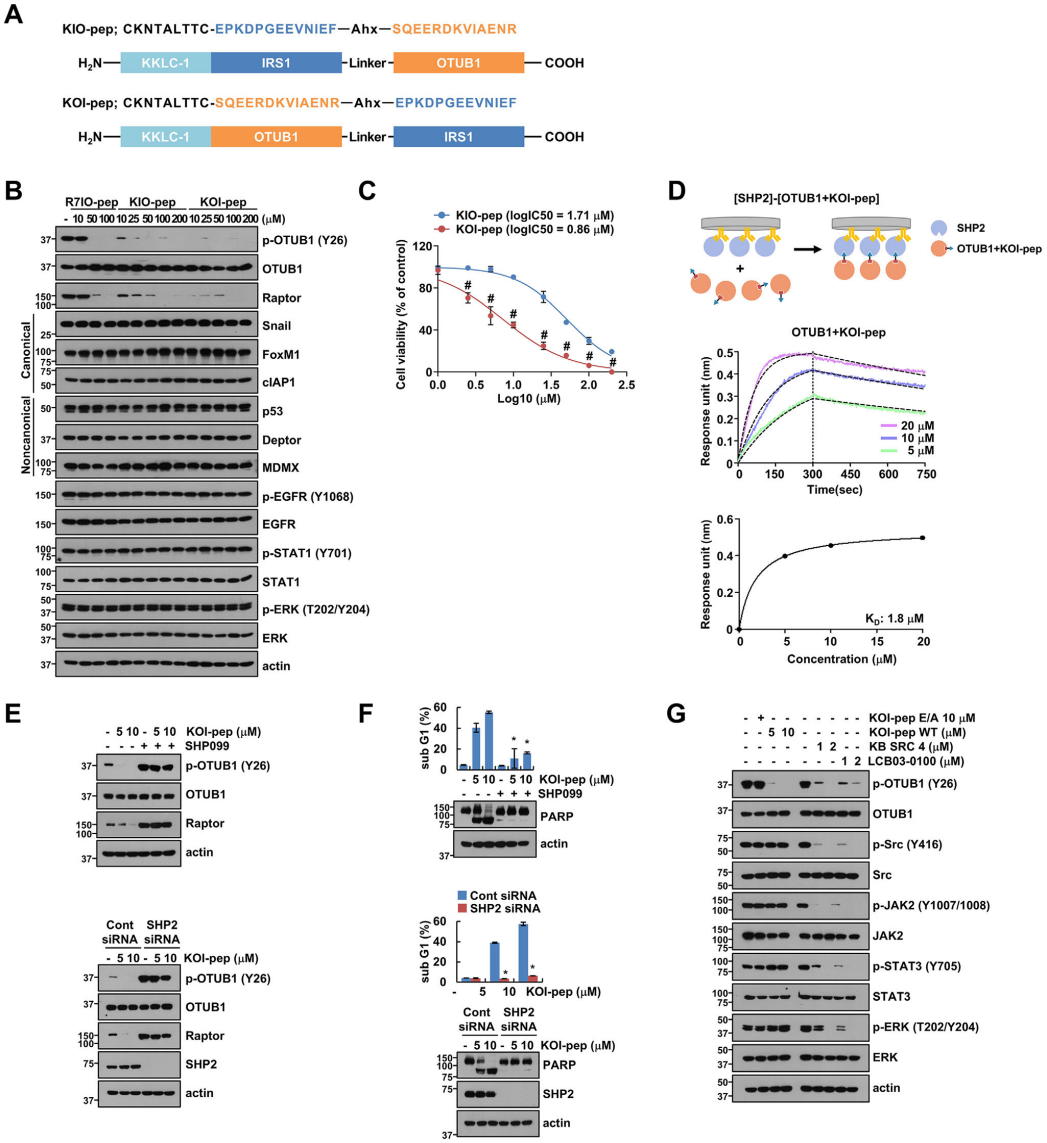
KKLC‐1, a tumor‐homing peptide, enhances the anti‐cancer effect of bioPhosTAC. (A) Schematic view of the designed bioPhosTAC according to peptide directionality. (B) Western blotting of protein expression in MDA‐MB231 cells treated with 10–200 µM R7IO‐pep, KIO‐pep, or KOI‐pep for 24 h. (C) MTT assay for cell viability in MDA‐MB231 cells treated with 1–200 µM KOI‐pep or KOI‐pep for 24 h. (D) BLI experiment for ternary complex formation (OTUB1‐KOI‐pep‐SHP2). (E,F) Western blotting (E,F) of protein expression and flow cytometry analysis (F) of the sub‐G1 population in MDA‐MB231 cells pretreated with 10 µM SHP099 (upper panel) or transfected with the indicated siRNAs (lower panel) followed by 5–10 µM KOI‐pep treatment for 24 h. (G) Western blotting of protein expression in MDA‐MB231 cells treated with KOI‐peptide or SRC inhibitors (KB SRC4 and LCB03‐0100) for 24 h. Values shown in the graphs represent the mean ± SD of results from three independent experiments. ^*^ *p* <0.05 compared with KOI‐pep; ^#^p < 0.05 compared with KIO‐pep.

In terms of concentration‐dependent activity of the three constructs, the KKLC‐1‐conjugated constructs showed markedly higher activity than that of the R7‐based peptide. Notably, the KKLC1–OTUB1–IRS1 (KOI‐pep) arrangement displayed a greater potency than that of the KKLC1–IRS1–OTUB1 (KIO‐pep) configuration, indicating that both improved uptake and proper module orientation are critical for effective dephosphorylation activity (Figure 3B). As a downstream consequence of OTUB1 Y26 dephosphorylation, KOI‐pep selectively reduced the levels of Raptor while sparing other OTUB1 canonical substrates (Snail, FoxM1, and cIAP1) and Y26‐independent noncanonical substrates (p53, Deptor, and MDMX). Importantly, none of the bioPhosTAC peptides altered the tyrosine phosphorylation of established SHP2 substrates, such as EGFR, STAT1, and ERK (Figure 3B), indicating that their activity is selective and does not impact unrelated phosphorylation events. To further determine whether KOI‐pep broadly perturbs canonical OTUB1 substrate regulation, we compared its effects with those of wild‐type (WT) OTUB1 overexpression and its catalytically inactive C91S mutant. As expected, overexpression of OTUB1 C91S dysregulated the representative canonical OTUB1 substrates. In contrast, KOI‐pep did not measurably affect either representative canonical OTUB1 substrates or Y26‐independent noncanonical OTUB1 substrates, despite efficiently reducing Raptor levels (Figure S2A). These results further support that KOI‐pep selectively modulates the Y26 phosphorylation‐dependent noncanonical function of OTUB1 without broadly perturbing canonical substrate regulation or other noncanonical OTUB1 signaling pathways. Moreover, we evaluated key mTORC1 downstream markers associated with translational regulation. KOI‐pep reduced the levels of phospho‐mTOR (S2448), phospho‐p70S6K, and phospho‐4EBP1 in a concentration‐dependent manner, which closely correlated with the reduction of Raptor expression (Figure S2B).

To examine the anti‐cancer efficacy of two peptides, we performed a dose‐response analysis. KOI‐pep decreased cell viability more potently than KIO‐pep, exhibiting a significantly lower logIC50 value of 0.86 µM compared to 1.71 µM for KIO‐pep (Figure 3C). To experimentally assess ternary complex formation, we conducted BLI analyses with SHP2 immobilized on the sensor surface (Figure 3D). Notably, the pre‐assembled OTUB1–bioPhosTAC complex displayed a distinct binding profile compared with bioPhosTAC alone. Following subtraction of non‐specific binding signals obtained from control sensors without SHP2 and correction for the contribution of bioPhosTAC alone, a substantially increased binding response was observed for the OTUB1–bioPhosTAC complex (Figure 3D). The corresponding control BLI experiments used for the ternary binding analysis are shown in Figure S2C. These findings provide biophysical evidence supporting the formation of an OTUB1–bioPhosTAC–SHP2 ternary complex. To further validate the SHP2 dependency of KOI‐pep, we examined its dephosphorylation activity under SHP2 inhibition or knockdown. As expected, KOI‐pep failed to induce OTUB1 Y26 dephosphorylation in the presence of the SHP2 inhibitor, SHP099, or when transfected with SHP2‐targeting siRNA, and Raptor levels were correspondingly restored (Figure 3E). As KOI‐pep had shown signs of reducing the viability of MDA‐MB231 cells (Figure 3C), we also evaluated the association between these effects and apoptosis. KOI‐pep induced apoptosis in a SHP2‐dependent manner, supporting that SHP2 recruitment is essential not only for OTUB1 Y26 dephosphorylation but also its downstream biological consequences (Figure 3F). To further evaluate the specificity of KOI‐pep‐mediated OTUB1 Y26 dephosphorylation, we compared its dephosphorylation activity with that of known Src kinase inhibitors (KB SRC 4 and LCB03‐011) because Src is an established endogenous kinase responsible for OTUB1 Y26 phosphorylation [10]. As expected, the Src inhibitors reduced tyrosine phosphorylation across multiple substrates, including JAK2, STAT3, ERK, and OTUB1. In contrast, KOI‐pep selectively reduced OTUB1 Y26 phosphorylation without affecting the other Src‐regulated substrates (Figure 3G). This selective dephosphorylation of OTUB1 Y26 highlights the fact that bioPhosTAC achieves a level of substrate specificity not observed with conventional kinase inhibitors. To further evaluate the selectivity of KOI‐pep toward OTUB1 Y26‐dependent signaling [7, 10], we examined its impact on interaction between OTUB1 and several OTUB1‐associated E2 enzymes. KOI‐pep selectively disrupted the interaction of OTUB1 with UBC13, UBC16, and UBCH5A, which are known to be regulated by Y26 phosphorylation, whereas no significant effects were observed for other OTUB1‐associated E2 enzymes (Figure S2D). Consistently, the OTUB1 Y26A mutant displayed markedly reduced binding affinity for UBC13, UBC16, and UBCH5A compared to the OTUB1 WT, supporting the importance of Y26 phosphorylation in mediating these non‐canonical OTUB1‐E2 interactions.

### 2.4 KOI‐pep Specifically Undergoes Clathrin‐Dependent Endocytosis in Cancer Cells

We investigated whether the markedly enhanced activity of KKLC‐1‐conjugated bioPhosTAC was linked to the expression profile of KKLC‐1 across different cell types. As expected, KKLC‐1 protein and mRNA were highly expressed in various cancer cell lines, including MDA‐MB231, A549, Caki, and HCT116, but showed low expression in normal cells, such as TCMK‐1, MC, and EA.hy926 (Figure 4A). Consistent with its proposed role as a targeting receptor, non‐permeabilized flow cytometric analysis confirmed cell‐surface localization of KKLC‐1 in MDA‐MB231 cells (Figure S3A). In addition, subcellular fractionation followed by immunoblotting demonstrated enrichment of KKLC‐1 in the membrane fraction (Figure S3B).

**FIGURE 4:**
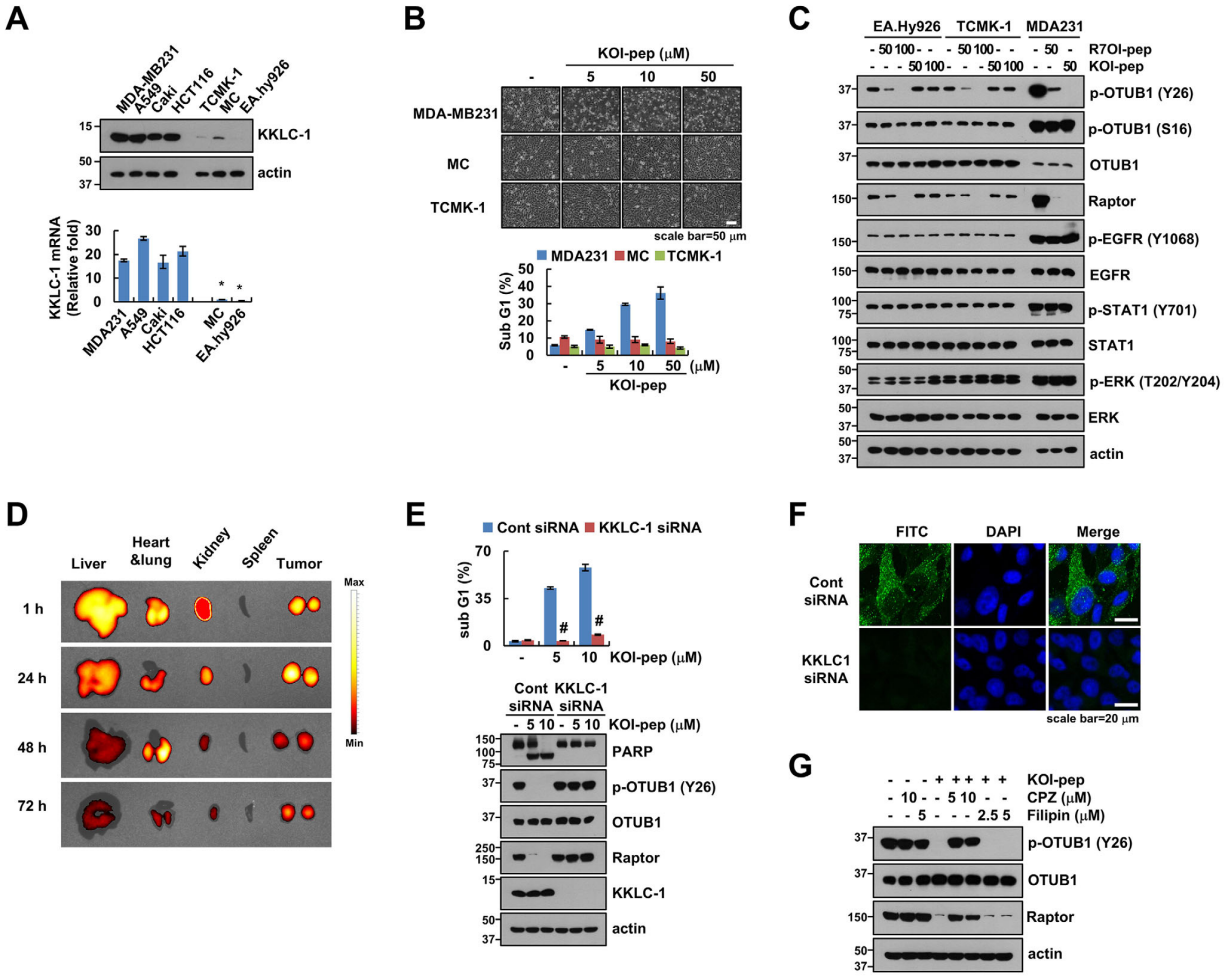
KOI‐peptide selectively increases cancer apoptosis through clathrin‐dependent endocytosis. (A) Western blotting of protein expression and qPCR analysis of mRNA expression in cancer (MDA‐MB231, A549, Caki, and HCT116) or normal (TCMK‐1, MC, and EA.hy926) cells. (B) Representative images and flow cytometry analysis of the sub‐G1 population in cancer (MDA‐MB231) or normal (MC and TCMK‐1) cells treated with 5–50 µM KOI‐pep treatment for 24 h. (C) Western blotting of protein expression in cancer (MDA‐MB231) or normal (EA.hy926 and TCMK‐1) cells treated with 50–100 µM KOI‐pep or R7OI‐pep for 24 h. (D) Representative in vivo fluorescence imaging of harvested major organs and tumor tissue showing the biodistribution and selective accumulation of KOI‐WT‐Cy5‐pep. (E) Flow cytometry analysis of the sub‐G1 population and western blotting of protein expression in MDA‐MB231 cells transfected with control or KKLC‐1 siRNA followed by 5–10 µM KOI‐pep treatment for 24 h. (F) Confocal micrographs showing the internalization of peptide in MDA‐MB231 cells transfected with control or SHP2 siRNA followed by 10 µM FITC‐labeled KOI‐pep treatment for 9 h. (G) Western blotting of protein expression in MDA‐MB231 cells pretreated with 5–10 µM chlorpromazine (CPZ) or 2.5–5 µM filipin followed by 10 µM KOI‐pep treatment for 24 h. Values in the graphs represent the mean ± SD of results from three independent experiments. ^*^ *p* < 0.05 compared with the cancer cells lines; #p < 0.05 compared with KOI‐pep.

To determine whether this expression pattern corresponded to biological effects of the optimized construct, we then evaluated apoptosis induction across these cell lines. KOI‐pep induced apoptosis in MDA‐MB231 cells in a concentration‐dependent manner, whereas no apoptotic response was observed in TCMK‐1 or MC cells (Figure 4B). We compared the cellular responses to R7IO‐pep and KOI‐pep in both normal and cancer cell lines to examine the contribution of KKLC‐1 targeting to functional selectivity. R7IO‐pep induced OTUB1 Y26 dephosphorylation and downregulation of Raptor expression in both normal and cancer cell lines at 50 µM, whereas KOI‐pep only induced these in cancer cell lines. Neither bioPhosTAC affected OTUB1 S16 phosphorylation nor altered the phosphorylation status of other examined proteins (Figure 4C).

To further evaluate the tumor‐targeting kinetics and stability of the KOI‐pep, we performed in vivo biodistribution analysis using Cy5‐labeled KOI‐pep in tumor‐bearing mice. In vivo fluorescence imaging revealed that KOI‐Cy5‐pep predominantly localized to the tumor sites identified by bioluminescence (Figure S3C). Furthermore, we investigated fluorescence imaging of harvested major organs and tumors to evaluate the organ‐specific biodistribution over an extended period. Initially, high fluorescence intensity was detected in vital organs (such as the liver, heart, lung, and kidney) at 1 h, whereas these signals significantly diminished over 48 h (Figure 4D). In contrast, KOI‐Cy5‐pep signal within the tumor tissue remained robust and clearly detectable even up to 72 h. These findings demonstrate that KOI‐pep possesses in vivo stability and selective tumor‐targeting capability. Consistent with these findings, KOI‐pep‐induced OTUB1 Y26 dephosphorylation, Raptor downregulation, and cell death were all suppressed upon KKLC‐1 knockdown (Figure 4E).

Furthermore, we confirmed the KKLC‐1‐dependent internalization of KOI‐pep via fluorescence microscopy. As expected, FITC‐labeled KOI‐pep successfully entered KKLC‐1‐positive MDA‐MB231 cells but failed to be internalized by KKLC‐1‐knockdown MDA‐MB231 cells, indicating that KOI‐pep relies on KKLC‐1‐mediated cellular uptake (Figure 4F). To elucidate the mechanism underlying this uptake, we examined the roles of two major endocytic pathways, clathrin‐ and caveolae‐mediated endocytosis, using specific inhibitors. As shown in Figure 4G, chlorpromazine (CPZ), an inhibitor of clathrin‐mediated endocytosis, markedly suppressed KOI‐pep‐induced OTUB1 Y26 dephosphorylation and restored Raptor expression. In contrast, filipin, a caveolin‐dependent endocytosis inhibitor, had no effect, thus indicating that clathrin uptake is required for KOI‐pep activity (Figure 4G). Collectively, these results demonstrate that KKLC‐1‐driven cellular uptake of KOI‐pep primarily occurs through clathrin‐dependent, rather than caveolae‐dependent, endocytosis.

### 2.5 KOI‐pep Ubiquitinates and Destabilizes Raptor Expression

To confirm whether the phosphotyrosine‐derived residue within the IRS1 peptide is important for SHP2 engagement, we generated a loss‐of‐function control in which the phosphomimetic glutamic acid (E) was substituted with alanine (A) (Figure 5A). This E→A substitution abolished the activity of KOI‐pep, thereby preventing OTUB1 Y26 dephosphorylation, Raptor downregulation, and KOI‐pep‐mediated apoptosis (Figure 5B). Accordingly, this inactive variant (KOI‐pep E/A) was used as a negative control in subsequent experiments.

**FIGURE 5:**
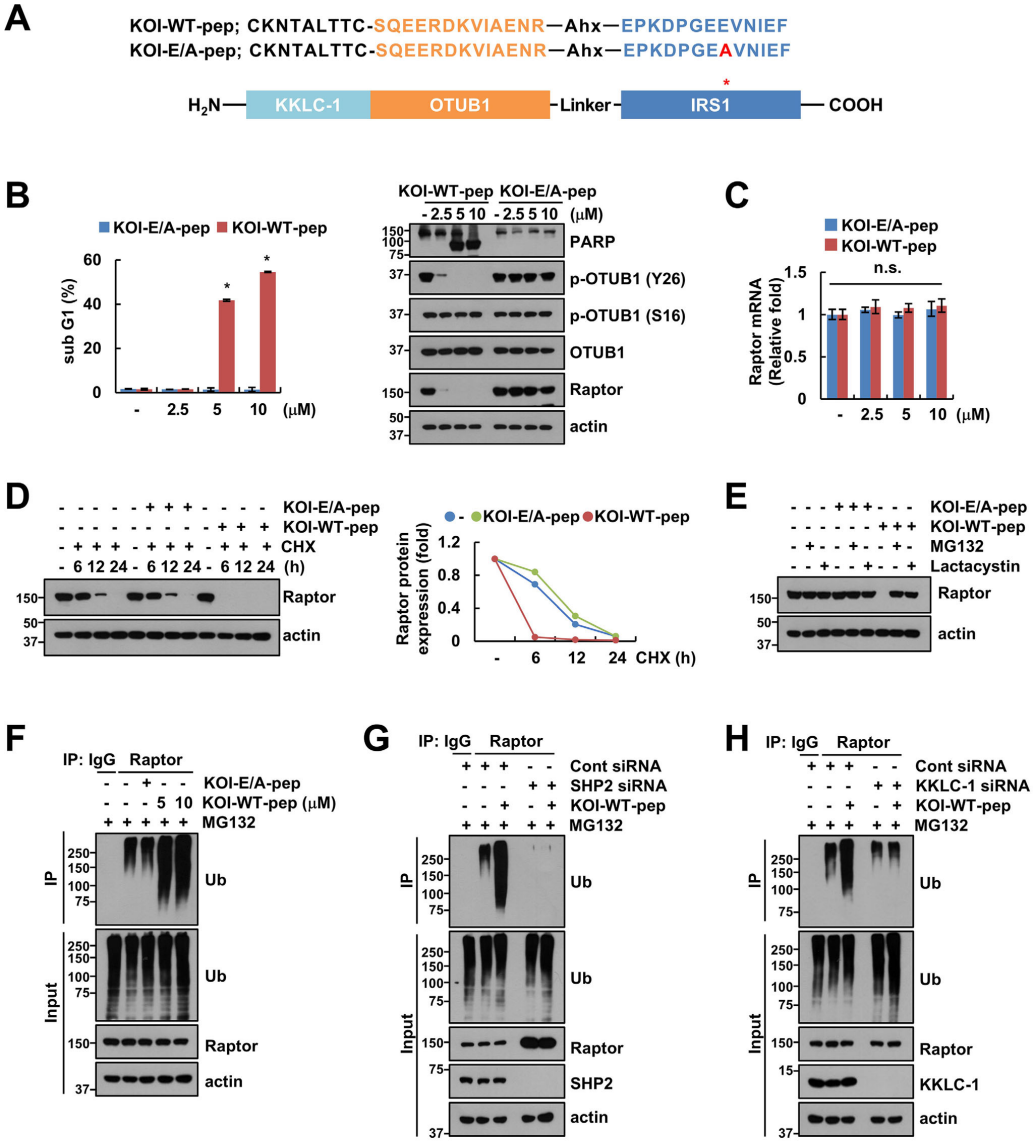
KOI‐pep degrades Raptor protein levels through proteasome activation. (A) Schematic view of the designed bioPhosTAC according to IRS mutation. (B) Flow cytometry analysis of the sub‐G1 population and western blotting of protein expression in MDA‐MB231 cells treated with 2.5–10 µM KOI‐WT‐pep or KOI‐E/A‐pep treatment for 24 h. (C) The qPCR results of mRNA expression in MDA‐MB231 cells treated with 2.5–10 µM KOI‐WT‐pep or KOI‐E/A‐pep treatment for 24 h. (D) Western blotting and quantification of protein expression in MDA‐MB231 cells pretreated with 20 µg/mL cycloheximide (CHX) followed by 10 µM KOI‐WT‐pep or KOI‐E/A‐pep treatment for the indicated times. (E) Western blotting of protein expression in MDA‐MB231 cells pretreated with 0.5 µM MG132 or 2.5 µM lactacystin followed by 10 µM KOI‐WT‐pep or KOI‐E/A‐pep treatment for 24 h. (F) Ubiquitination assay of ubiquitinated Raptor in MDA‐MB231 cells transfected with HA‐Ub followed by 10 µM KOI‐WT‐pep or KOI‐E/A‐pep treatment with MG132. (G,H) Ubiquitination assay of ubiquitinated Raptor in MDA‐MB231 cells transfected with control, SHP2 siRNA (G) or KKLC‐1 siRNA (H), and HA‐Ub followed by 10 µM KOI‐WT‐pep treatment with MG132. Values in the graphs represent the mean ± SD of results from three independent experiments. ^*^ *p* < 0.05 compared with the control.

Our previous work established that Raptor stability is regulated at the protein level via phosphorylation of OTUB1 at Y26, rather than at the transcriptional level [10]. Based on this framework, we examined whether KOI‐pep modulates Raptor through a similar post‐translational mechanism driven by selective OTUB1 Y26 dephosphorylation. Consistent with this model, KOI‐pep did not alter Raptor mRNA expression (Figure 5C). Instead, Raptor protein expression was selectively downregulated by KOI‐WT‐pep and exhibited accelerated degradation in the presence of the protein synthesis inhibitor, cycloheximide, an effect that was reversed by the proteasome inhibitors, MG132 and lactacystin (Figure 5D,E). Consistently, KOI‐WT‐pep, but not the inactive KOI‐E/A‐pep mutant, increased Raptor ubiquitination, indicating that KOI‐pep promotes the proteasome‐dependent degradation of Raptor (Figure 5F). Furthermore, Raptor ubiquitination was abolished under SHP2 or KKLC‐1 knockdown, confirming that KOI‐pep‐mediated Raptor destabilization requires both SHP2 recruitment and KKLC‐1‐dependent internalization (Figure 5G,H). Together, these results demonstrate that KOI‐pep regulates Raptor through a post‐translational degradation mechanism that specifically arises from OTUB1 Y26 dephosphorylation.

Additionally, we generated a Binder_13_ triple mutant (Q2A/R5A/R13A) designed to abolish OTUB1 binding based on the AlphaFold‐predicted interaction interface (Figure S4A). As expected, each individual mutant exhibited reduced binding affinity toward OTUB1 (Figure S4B). Using the KOI‐OTU Mut‐pep construct, we further evaluated downstream biological effects including OTUB1 Y26 dephosphorylation, Raptor downregulation, and apoptosis induction, analogous to the analyses previously performed with the KOI‐E/A mutant (Figure S4C). Collectively, these findings demonstrate that the Binder_13_ sequence is required for OTUB1 engagement and is essential for bioPhosTAC activity.

### 2.6 KOI‐pep Increases Mitochondrial ROS Levels and Fusion

Loss of Raptor resulting from impaired OTUB1 Y26 signaling leads to increased mitochondrial reactive oxygen species (ROS) production and altered mitochondrial fusion dynamics [10]. Based on this established link between the OTUB1–Raptor axis and mitochondrial homeostasis, we then examined whether KOI‐WT‐pep induces similar mitochondrial phenotypes with its negative control (KOI‐E/A‐pep). KOI‐WT‐pep selectively increased mitochondrial ROS production, as indicated by the enhanced pHyPer‐dMito fluorescence observed, whereas nuclear or cytosolic ROS levels remained unchanged (Figure 6A). In contrast, the KOI‐E/A‐pep mutant failed to induce mitochondrial ROS production, supporting that KOI‐WT‐pep perturbs mitochondrial function through the dephosphorylation of Y26 on OTUB1. Consistently, KOI‐WT‐pep, but not KOI‐E/A‐pep, elevated MitoSOX Red fluorescence (Figure 6B), confirming that KOI‐WT‐pep specifically triggers mitochondrial superoxide production.

**FIGURE 6:**
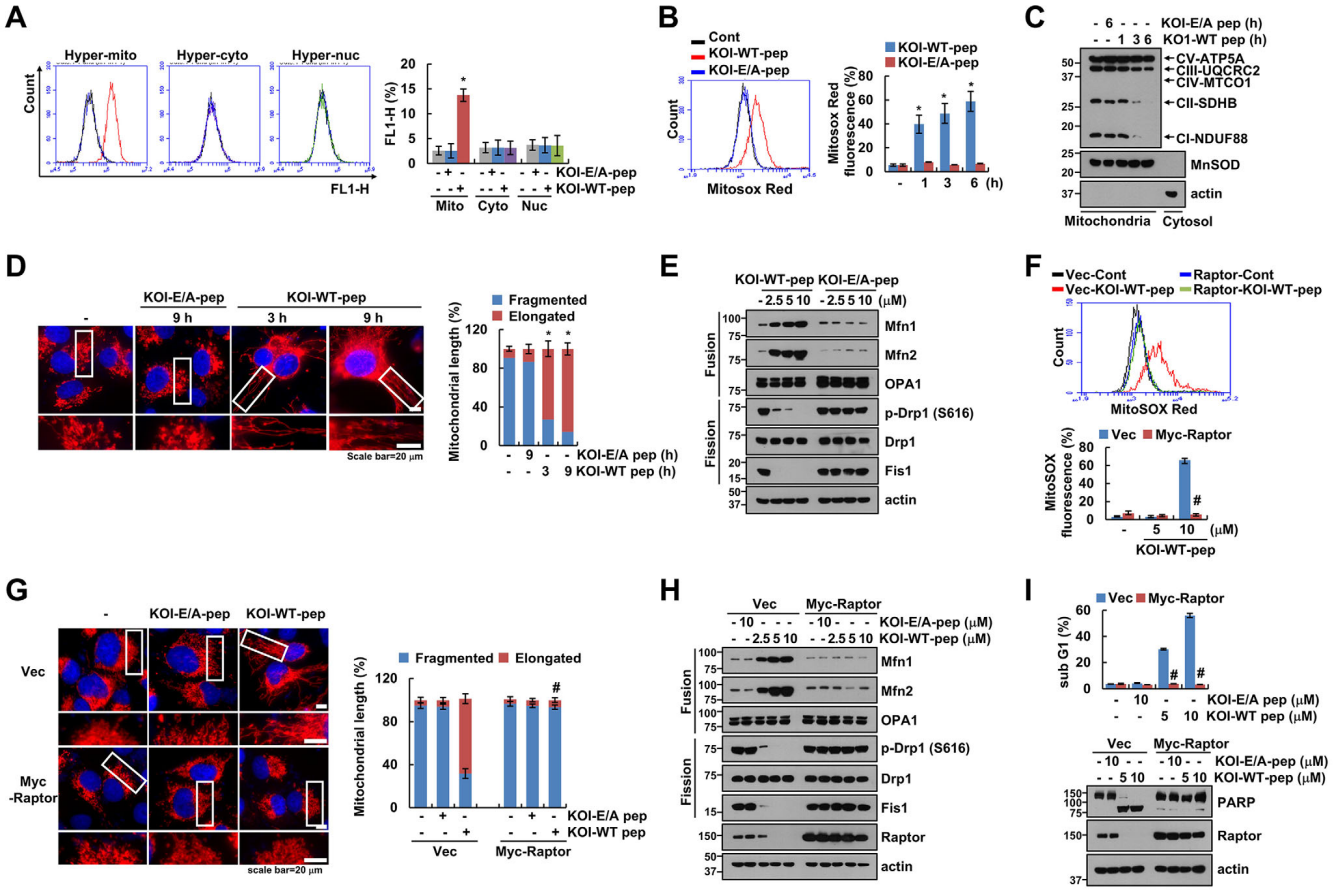
KOI‐pep induces mitochondrial fusion and ROS generation through Raptor degradation. (A) Flow cytometry analysis of ROS generation in MDA‐MB231 cells transfected with Hyper‐mito, Hyper‐cyto, or Hyper‐nuc plasmids followed by 10 µM KOI‐WT‐pep or KOI‐E/A‐pep treatment for 6 h. (B) Flow cytometry analysis of mitochondrial ROS levels using MitoSOX Red dye in MDA‐MB231 cells treated with 10 µM KOI‐WT‐pep or KOI‐E/A‐pep treatment for 6 h. (C) Western blotting of protein expression using the mitochondrial fraction in MDA‐MB231 cells treated with 10 µM KOI‐WT‐pep or KOI‐E/A‐pep treatment for the indicated times. (D) Fluorescence micrographs and quantification of mitochondrial length in Caki‐1/pDsRed2‐Mito cells treated with 10 µM KOI‐WT‐pep or KOI‐E/A‐pep treatment for the indicated times. (E) Western blotting of protein expression in MDA‐MB231 cells treated with 2.5–10 µM KOI‐WT‐pep or KOI‐E/A‐pep treatment for 24 h. (F) Flow cytometry analysis of mitochondrial ROS levels using MitoSOX Red dye in MDA‐MB231 cells transfected with vector or myc‐Raptor plasmids followed by 5–10 µM KOI‐pep treatment for 6 h. (G) Fluorescence micrographs and quantification of mitochondrial length in Caki‐1/pDsRed2‐Mito cells transfected with vector or myc‐Raptor plasmids followed by 5–10 µM KOI‐pep treatment for 6 h. (H) Western blotting of protein expression in MDA‐MB231 cells transfected with vector or myc‐Raptor plasmids followed by 2.5–10 µM KOI‐WT‐pep or 10 µM KOI‐E/A‐pep treatment for 24 h. (I) Flow cytometry analysis of the sub‐G1 population and western blotting of protein expression in MDA‐MB231 cells transfected with vector or myc‐Raptor plasmids, followed by 2.5–10 µM KOI‐WT‐pep or 10 µM KOI‐E/A‐pep treatment for 24 h. Values shown in the graphs represent the mean ± SD of results from three independent experiments. ^*^ *p* < 0.05 compared with the control; #p < 0.05 compared with KOI‐WT‐pep.

We examined mitochondrial respiratory complexes. KOI‐WT‐pep downregulated OXPHOS complexes I (NDUFB8), II (SDHB), and III (UQCRC2) (Figure 6C), providing a mechanistic basis for KOI‐pep‐induced mitochondrial ROS generation. Accordingly, KOI‐WT‐pep promoted mitochondrial fusion, whereas KOI‐E/A‐pep had no effect (Figure 6D). Mitochondrial dynamics regulators were also altered upon KOI‐WT‐pep treatment. KOI‐WT‐pep increased expression of the fusion mediators, Mfn1 and Mfn2, while reducing that of the fission regulators, Fis1 and phospho‐Drp1 (S616) (Figure 6E). These changes were absent in KOI‐E/A‐pep‐treated cells, indicating that functional OTUB1 Y26 dephosphorylation is required to drive mitochondrial remodeling. To determine whether Raptor mediates these mitochondrial defects, we ectopically expressed Raptor and assessed mitochondrial phenotypes following KOI‐pep exposure. Raptor overexpression markedly suppressed KOI‐pep‐induced mitochondrial ROS production, fusion (Figure 6F–H), and apoptosis (Figure 6I). Together, these data demonstrate that Raptor acts as a key downstream effector following KOI‐pep treatment, mediating mitochondrial dysfunction and cell death in response to OTUB1 Y26 dephosphorylation.

As KOI‐pep disrupted mitochondrial homeostasis through selective OTUB1 Y26 dephosphorylation, we then assessed whether this mitochondrial phenotype was dependent on SHP2 recruitment and KKLC‐1‐mediated uptake. Pretreatment with SHP099 or SHP2 siRNA markedly reduced KOI‐WT‐pep‐induced mitochondrial ROS production and fusion, including the associated upregulation of Mfn1/2 and downregulation of Fis1 and phospho‐Drp1 (S616) (Figure S5A–F). Consistent with the requirement for KKLC‐1‐mediated uptake, KKLC‐1 knockdown similarly attenuated KOI‐WT‐pep activity (Figure S5G–I). As KOI‐pep enters cells through clathrin‐dependent endocytosis, which is essential for its ability to reduce Raptor levels (Figure 4F), we subsequently examined whether this uptake mechanism also influenced mitochondrial responses. Indeed, CPZ effectively suppressed KOI‐WT‐pep‐induced mitochondrial ROS production and fusion (Figure S5J,K). Furthermore, CPZ reversed the KOI‐WT‐pep‐driven increases in fusion markers (Mfn1/2) and decreases in fission markers (phospho‐Drp1 and Fis1), whereas filipin exhibited no such effect (Figure S5L). Together, these results indicate that SHP2 engagement and KKLC‐1‐dependent, clathrin‐mediated internalization are required for KOI‐pep‐induced mitochondrial fusion and ROS generation through OTUB1 Y26 dephosphorylation‐dependent Raptor degradation (Figure S5). Importantly, pharmacological inhibition or knockdown of SHP2 abolished KOI‐pep‐induced mitochondrial dysfunction, indicating that this phenotype is dependent on SHP2‐mediated bioPhosTAC activity (Figure S5C,F).

### 2.7 KOI‐pep Activates the OTUB1–Raptor–Bim Signaling Axis to Induce Apoptosis and Suppress Tumor Growth In Vivo

Our previous study showed that the degradation of Raptor‐mediated mitochondrial dysfunction upregulates Bim expression, a pro‐apoptotic Bcl‐2 family protein, thereby increasing sensitivity to anti‐cancer drug‐mediated apoptosis [26]. Based on this, we examined the contribution of Bim to KOI‐pep‐mediated apoptosis. As expected, KOI‐WT‐pep increased Bim expression (Figure 7A).

**FIGURE 7:**
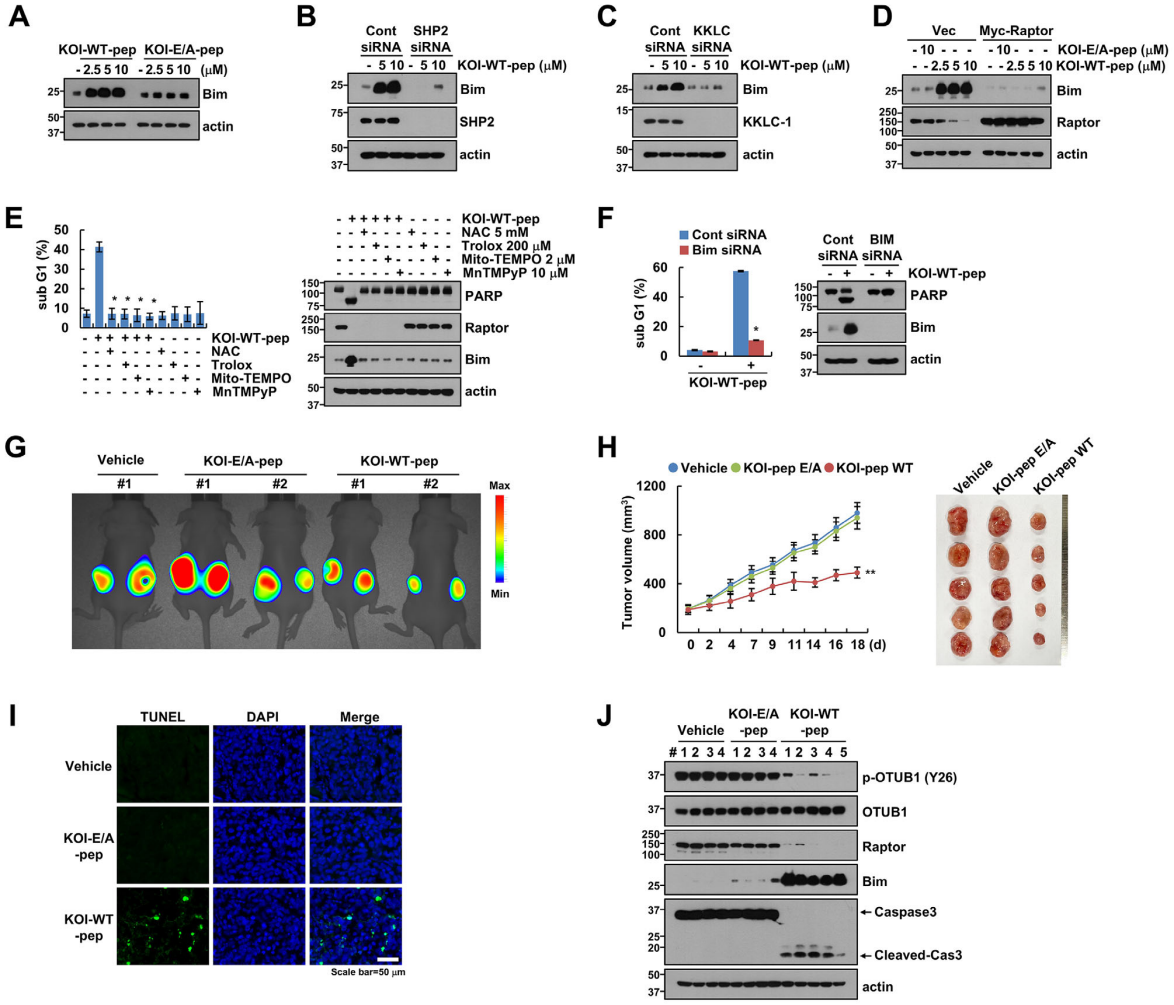
KOI‐pep increases apoptosis through the SHP2–OTUB1 Y26–Raptor–Bim axis in vitro and in vivo. (A) Western blotting of protein expression in MDA‐MB231 cells treated with 5–10 µM KOI‐WT‐pep or KOI‐E/A‐pep treatment for 24 h. (B,C) Western blotting of protein expression in MDA‐MB231 cells transfected with control, SHP2 (B), or KKLC‐2 (C) siRNA followed by 5–10 µM KOI‐WT‐pep treatment for 24 h. (D) Western blotting of protein expression in MDA‐MB231 cells transfected with the vector or Myc‐Raptor followed by 5–10 µM KOI‐WT‐pep or KOI‐E/A‐pep treatment for 24 h. (E) Flow cytometry analysis of the sub‐G1 population and western blotting of protein expression and in MDA‐MB231 cells pretreated with a ROS scavenger followed by 10 µM KOI‐WT‐pep treatment for 24 h. (F) Flow cytometry analysis of the sub‐G1 population and western blotting of protein expression in MDA‐MB231 cells transfected with control or Bim siRNA followed by 10 µM KOI‐WT‐pep treatment for 24 h. (G–J) Representative in vivo bioluminescence imaging (G), tumor growth (H), TUNEL staining (I), and western blotting analysis (J) of the 10 mg/kg KOI‐WT‐pep‐ or KOI‐E/A‐pep‐treated MDA‐MB231‐Luc xenograft model established in BALB/c‐nude mice. Values in the graphs represent the mean ± SD of results from three independent experiments. ^*^ *p* < 0.05 compared with KOI‐WT‐pep; ^**^ *p* < 0.05 compared with the vehicle.

However, this upregulation was abrogated by the silencing of SHP2 or KKLC‐1, as well as by the ectopic expression of Raptor (Figure 7A–D). Global ROS (*N*‐acetyl‐l‐cysteine and Trolox) and mitochondrial ROS (Mito‐TEMPO and MnTMPyP) scavengers completely blocked KOI‐WT‐pep‐induced apoptosis (Figure 7E). Notably, these ROS scavengers did not restore KOI‐pep‐mediated Raptor downregulation (Figure 7E), indicating that Raptor is positioned upstream of the ROS‐generating axis. Consistent with this model, Bim knockdown significantly reduced KOI‐WT‐pep‐induced apoptosis (Figure 7F).

Having established that KOI‐pep induces apoptosis through the SHP2–OTUB1 Y26–Raptor axis and subsequent Bim upregulation in vitro, we then asked whether these effects translate in vivo. To evaluate the anti‐tumor efficacy of KOI‐pep in vivo, KOI‐WT‐pep was administered to a xenograft mouse model via intravenous tail‐vein injection. KOI‐WT‐pep markedly suppressed tumor growth, whereas the inactive KOI‐E/A mutant showed no difference when compared with the vehicle group (Figure 7G,H). Consistent with our cellular findings, KOI‐WT‐pep increased TUNEL‐positive signals in tumor tissues and downregulated Raptor expression through OTUB1 Y26 dephosphorylation, leading to enhanced Bim expression (Figure 7I,J). These effects were absent in tumors treated with KOI‐E/A‐pep. Collectively, these results demonstrate that KOI‐pep elicits anti‐tumor activity in vivo through the same SHP2–OTUB1 Y26–Raptor–Bim apoptotic pathway identified in vitro, supporting the potential of bioPhosTAC for targeted cancer therapy.

## 3 Discussion

In this study, we introduce bioPhosTAC, a peptide‐based proximity platform that enables the selective tyrosine dephosphorylation of endogenous proteins through recruitment of the phosphatase, SHP2. Although induced‐proximity strategies for dephosphorylation have recently emerged as a promising modality for signaling control, most systems reported to date have focused on serine/threonine phosphatases or relied on a narrow subset of tyrosine phosphatases, most notably PTPN2 [15, 27]. By contrast, bioPhosTAC provides a programmable, fully peptide‐based approach that can expand substrate scope and enable the tunable manipulation of tyrosine phosphorylation.

Importantly, bioPhosTAC enabled the selective dephosphorylation of OTUB1 at Y26, a residue previously identified as a key regulator of the noncanonical OTUB1–Raptor signaling axis (Figures 2B and 3B). Although prior studies have established that phosphorylation at Y26 stabilizes Raptor and contributes to mTORC1‐dependent survival pathways, the experimental strategies used to probe the mechanism, such as phosphomimetic mutants or kinase modulation, lack the precision and reversibility required for manipulating endogenous OTUB1 and, therefore, offer limited potential for therapeutic application [10]. By directly modulating the phosphorylation state of OTUB1 Y26 in living cells, bioPhosTAC provides an innovative approach for the selective and reversible manipulation of this modification, enabling a more definitive dissection of its functional roles. Notably, these findings highlight endogenous, site‐specific perturbation of OTUB1 Y26 as the potential relationship between OTUB1 phosphorylation and cancer‐associated metabolic decisions.

One important implication of this work is that, until now, no experimental strategy to selectively modulate the noncanonical function of OTUB1 at the endogenous protein level. Previous approaches relied on genetic perturbation or upstream kinase inhibition, both of which inevitably affect multiple signaling axes and do not allow functional separation between the catalytic deubiquitinase activity of OTUB1 and its phosphorylation‐dependent regulatory roles (Figure 3F). By selectively editing the phosphorylation state of OTUB1 at Y26, bioPhosTAC represents the first tool for directly interrogating and manipulating this noncanonical signaling branch without interfering with canonical ubiquitin hydrolysis. These findings establish OTUB1 Y26 as a regulatory node that can be dynamically and reversibly controlled, rather than as a static post‐translational modification inferred from mutational analyses.

Mechanistically, our data support a model in which bioPhosTAC activity is driven by productive ternary complex formation rather than nonspecific phosphatase activation. Using both BLI and co‐immunoprecipitation assays, we confirmed the formation of an OTUB1–bioPhosTAC–SHP2 ternary complex (Figures 2E and 3D). Furthermore, disruption of either the OTUB1‐binding module or the SHP2‐recruiting module through rationally designed mutations abolished ternary complex formation and consequently eliminated OTUB1 Y26 dephosphorylation (Figure 5 and Figure S4). These observations establish that productive proximity between SHP2 and OTUB1 is an essential mechanistic requirement for bioPhosTAC activity. Similar to the cooperativity‐dependent mechanisms observed in other induced‐proximity technologies such as PROTACs, our findings demonstrate that biological activity is directly linked to ternary complex assembly rather than the individual binding properties of each module alone.

Another conceptual advance emerging from this study is that proximity can be rationally engineered using artificially designed peptides, rather than by relying on naturally evolved protein–protein interactions or pre‐existing small‐molecule ligands. The identification of an OTUB1‐binding peptide through de novo design, combined with a phosphatase‐recruiting module, demonstrates that proximity‐inducing systems can be modularly assembled even for targets lacking known binders [28, 29]. In this context, bioPhosTAC represents more than just a single tool for OTUB1 regulation; it illustrates a generalizable framework in which binding specificity and enzymatic recruitment can be independently optimized and recombined. This flexibility is particularly relevant for signaling proteins whose regulation depends on site‐specific post‐translational modifications.

Beyond OTUB1, induced proximity‐based dephosphorylation may be broadly applicable to phospho‐dependent signaling pathways that are difficult to access using conventional inhibitors. Tyrosine phosphorylation is typically regulated by kinases and phosphatases with broad substrate repertoires, complicating selective intervention [30, 31]. By restricting phosphatase activity through spatial proximity rather than catalytic inhibition, bioPhosTAC achieves superior levels of substrate and site selectivity over conventional pharmacological alternatives. The mode of action is fundamentally different from that of global kinase inhibition and may reduce unintended pathway rewiring, which limits therapeutic applicability. Notably, the peptide‐based nature of bioPhosTAC raises interesting possibilities for future development [32]. Although peptides are often considered as challenging therapeutic modalities, advances in delivery, stabilization, and targeting sequences—as exemplified by the KKLC‐1 tumor‐homing module used in the present study—are rapidly expanding their translational potential [33, 34]. Another notable feature of the bioPhosTAC platform is its built‐in tumor selectivity. Incorporation of the KKLC‐1‐derived tumor‐homing sequence [23, 24, 25] conferred preferential uptake into cancer cells via clathrin‐dependent endocytosis, while normal epithelial cells showed no change (Figure 4C). Such integrated control over biochemical specificity and cellular selectivity addresses a long‐standing limitation of peptide therapeutics and enhances the translational relevance of this approach. In this regard, bioPhosTAC may serve as both a mechanistic probe to dissect phospho‐regulated protein functions and a starting point for the development of next‐generation proximity‐based therapeutics that act through post‐translational signal editing rather than protein elimination.

From a mechanistic perspective, we demonstrated that selective manipulation of a single tyrosine phosphorylation site is sufficient to propagate coordinated cellular consequences. KOI‐pep‐mediated dephosphorylation of OTUB1 Y26 accelerated the proteasomal degradation of Raptor, disrupted mitochondrial oxidative phosphorylation, increased ROS generation, and ultimately triggered apoptosis (Figure 8).

**FIGURE 8:**
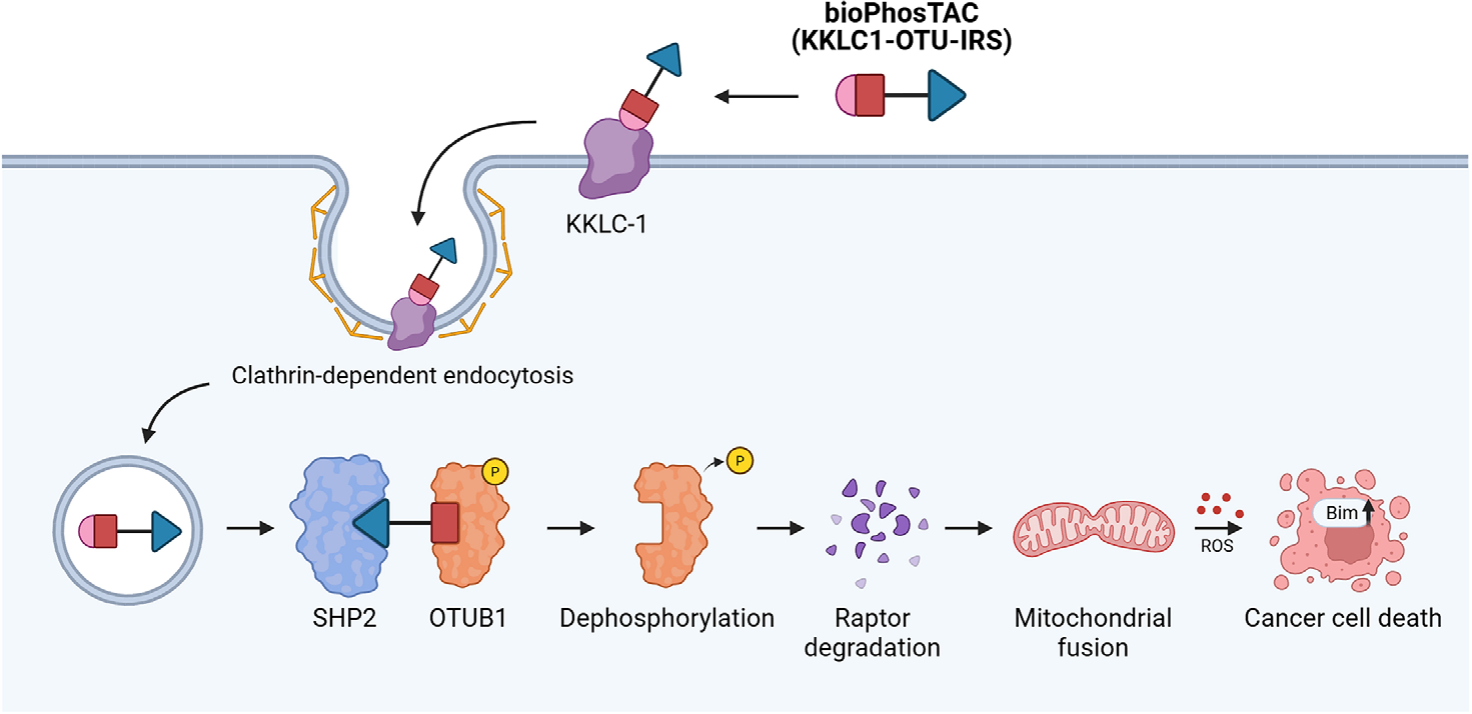
Scheme showing the anti‐tumor effect of bioPhosTAC‐mediated through the SHP2–OTUB1 Y26–Raptor axis.

This highlights how proximity‐induced dephosphorylation can function as a form of post‐translational signal editing, allowing discrete molecular interventions to reprogram complex cellular phenotypes. Beyond the specific OTUB1–SHP2 axis, our study provides broader implications for induced‐proximity technologies. The identification of a previously unreported OTUB1‐binding peptide illustrates how rational peptide engineering can generate recruiting modules for proteins that lack conventional ligands. This strategy is readily extensible and may facilitate the expansion of proximity‐based modulation to a wide range of challenging targets and post‐translational modifications.

Finally, the strong antitumor activity observed in both in vitro and in vivo models highlights the therapeutic potential of selectively modulating tyrosine phosphorylation when using our approach (Figure 7G–J). By enabling the direct and reversible editing of phosphorylation sites within endogenous proteins, bioPhosTAC provides a framework that can be adapted to interrogate diverse signaling nodes and potentially guide the design of next‐generation induced‐proximity therapeutics. More broadly, this work positions the versatile performance of the peptide‐based bioPhosTAC platform for dissecting phosphorylation‐dependent biology and expanding the scope of induced‐proximity technologies.

## 4 Experimental Section

### 4.1 Preparation of bioPhosTACs

All bioPhosTAC peptides were custom synthesized by DANDI CURE (Korea). Peptide purity and identity were confirmed by analytical HPLC and mass spectrometry. The corresponding quality‐control data provided by DANDI CURE (Korea) is included in Figure S6.

### 4.2 Protein Purification

OTUB1_40–271_ and SHP2 were cloned into the pParallelHIS2 vector and the pET‐28a vector, respectively. Binder_19_ and Binder_13_ were cloned into the pParallelGST2 vector [35]. For the expression of HIS_6_‐tagged OTUB1_40–271_ and SHP2, T7 Express *Escherichia coli* Competent cells (NEB, Ipswich, MA, USA) were transformed with these plasmids and grown in LB medium to an optical density at 600 nm (OD600) of 0.6–0.8 at 37°C. Protein production was induced through the addition of 0.1 mM isopropyl‐d‐thiogalactopyranoside(IPTG) for OTUB1_40–271_ and 0.5 mM IPTG for SHP2. The cells were further grown overnight at 16°C and collected. The cell pellet was resuspended in lysis buffer (50 mM Tris‐HCl, 150 mM NaCl, 20 mM imidazole, 1 mM TCEP, pH 8.0), lysed via sonication, and then centrifuged at 12,000 × *g* to clarify the supernatant. The supernatant was incubated for 2 h with NI SEPHAROSE 6FF (Cytiva, Marlborough, MA, USA) pre‐equilibrated with lysis buffer, and nonspecific proteins cleared with washing buffer (50 mM Tris‐HCl, 500 mM NaCl, 20 mM imidazole, 1 mM TCEP, pH 8.0). Proteins were eluted with elution buffer (50 mM Tris‐HCl, 500 mM NaCl, 300 mM imidazole, 1 mM TCEP, pH 8.0). The eluted proteins were buffer‐exchanged to IEX buffer A (20 mM Tris‐HCl, 20 mM NaCl, 1 mM TCEP, pH 8.0) using a centrifugal filter unit (molecular weight cutoff: 10 kDa (OTUB1_40–271_)/30 kDa (SHP2); Merck Millipore, Darmstadt, Germany) and purified via anion‐exchange chromatography on a HiTrap Q column (Cytiva) with gradient elution using IEX buffer B (20 mM Tris‐HCl, 1 M NaCl, 1 mM TCEP, pH 8.0). Selected proteins were loaded onto a size‐exclusion column (Superdex 75 Increase 10/300 GL; Cytiva) and pre‐equilibrated with SEC buffer (20 mM Tris‐HCl, 50 mM NaCl, 1 mM TCEP, pH 8.0). Proteins were stored for BLI. For OTUB1_40–271_, instead of using the elution buffer, Ni‐NTA beads were incubated with sfGFP‐TEV protease [36] for 2 h at 25°C and then overnight at 4°C. Similar for the eluted proteins described earlier, HIS_6_‐tagged cleaved proteins were buffer‐exchanged to IEX buffer A using a centrifugal filter unit and purified via anion‐exchange chromatography on a HiTrap Q column with gradient elution using IEX buffer B. Selected proteins were loaded onto a size‐exclusion column and pre‐equilibrated with SEC buffer. Proteins were stored for the ubiquitin chain cleavage assay. For the expression of GST‐Binder_19_, GST‐Binder_13_, and GST, BL21(DE3) *E. coli* Competent cells (NEB) were transformed with plasmids and grown in LB medium to an OD600 of 0.6–0.8 at 37°C. Protein production was induced through the addition of 0.5 mM IPTG. The cells were further grown overnight at 17°C and collected thereafter. The cell pellet was resuspended in lysis buffer (50 mM Tris‐HCl, 150 mM NaCl, 2 mM DTT, pH 8.0), lysed via sonication, and then centrifuged at 12,000 × *g* to clarify the supernatant. The supernatant was incubated for 2 h with glutathione Sepharose 4B (Cytiva), pre‐equilibrated with lysis buffer, and nonspecific proteins cleared with washing buffer (50 mM Tris‐HCl, 500 mM NaCl, 2 mM DTT, pH 8.0). Proteins were eluted with elution buffer (50 mM Tris‐HCl, 50 mM NaCl, 15 mM reduced glutathione, 2 mM DTT, pH 8.0). The eluted proteins were concentrated using a centrifugal filter unit, and selected proteins loaded onto a size‐exclusion column and pre‐equilibrated with SEC buffer. Proteins were stored for BLI. All purified proteins were evaluated their purity by SDS‐PAGE with Coomassie staining (Figure S1B).

### 4.3 Design of a Binder with RFdiffusion

60 Backbone models were initially generated with RFdiffusion (v.1.1.0), and a single backbone was selected for subsequent ProteinMPNN enrichment. Over 2000 sequences were explored by ProteinMPNN (v.1.0.1). Initial hits were screened using *dl_binder_design* tools, in which candidates with an interface pAE_interaction <6.0 Å were prioritized. The resulting complexes were further validated with AlphaFold2‐multimer (global pLDDT >85). In this framework, 9 peptide candidates were selected by the aggregate confidence (Table S1) (Supporting Information).

### 4.4 Biolayer Interferometry

Binding kinetics were determined with the GatorPrime system (Gator Bio, Palo Alto, CA, USA) at 30°C. His‐OTUB1_40–271_ at 10 µg/mL was loaded onto Ni‐NTA biosensors and equilibrated with binding buffer to measure the baseline. To examine the association rate, equilibrated sensors were transferred into solutions containing various concentrations of GST‐ Binder_19_ (2.5–10 µM), GST‐Binder_13_ (5–15 µM), GST‐ Binder_13_ (Q2A, R5A, R13A) (5–15 µM), GST (2.5–10 µM/5–15 µM). Dissociation of OTUB1 was initiated by placing the sensor into the reaction buffer. Binding responses obtained with GST alone were used as a reference and subtracted from those of GST‐Binder. To evaluate ternary complex formation, His‐SHP2 at 10 µg/mL was loaded onto Anti‐HIS biosensors and equilibrated with binding buffer to establish the baseline. OTUB1 at various concentrations (5–20 µM) was pre‐incubated with 50 µM PhosTAC for 30 min at room temperature, and the pre‐incubated OTUB1–PhosTAC mixtures were then applied as analytes to the SHP2‐loaded sensors. Dissociation was initiated by placing the sensors into reaction buffer. Binding responses obtained from sensors without immobilized SHP2, as well as responses from PhosTAC alone, were used as references and subtracted from the corresponding binding responses. Background‐corrected responses were analyzed by steady‐state fitting to estimate the apparent K*d* value using Gator Dator analysis software (GatorBio).

### 4.5 Cell Lines and Chemicals

Human cancer (MDA‐MB231, A549, Caki, and HCT116) and normal (kidney: TCMK‐1, human umbilical vein endothelial: EA.hy926, and mesangial cells: MC) cell lines were obtained from the American Type Culture Collection (Manassas, VA, USA). All cells were cultured in appropriate medium containing 10% fetal bovine serum (Hyclone, Logan, UT, USA), 1% penicillin‐streptomycin, and 100 µg/mL gentamycin (Thermo Fisher Scientific, Waltham, MA, USA). Trolox and MnTMPyP were obtained from Merck Millipore (Darmstadt, Germany). Lactacystin was purchased from Enzo Life Sciences (Ann Arbor, MI, USA). All other chemicals used were purchased from Sigma–Aldrich (St. Louis, MO, USA).

### 4.6 Western Blotting

Cells were lysed using radioimmunoprecipitation assay buffer containing protease inhibitors [37] and centrifuged at 13,000 × *g* at 4°C for 15 min to obtain proteins. Proteins were separated using SDS‐PAGE and transferred to nitrocellulose membranes (GE Healthcare Life Sciences, Pittsburgh, PA, USA). Protein expression was detected using an enhanced chemiluminescence kit (Cytiva, Buckinghamshire, United Kingdom). Information on the antibodies used in this study is provided in Table S2 (Supporting Information).

### 4.7 Apoptosis

Trypsinized cells were fixed using 95% ethanol at 4°C. After 1 h, the cells were resuspended in 1.12% sodium citrate buffer (pH 8.4) containing 12.5 µg RNase and incubated at 37°C for 30 min. After incubation, 250 µL propidium iodide solution (50 µg/mL) was added to the cells, followed by incubation at 37°C for 30 min. The number of apoptotic cells was measured using a BD Accuri C6 flow cytometer (BD Biosciences, San Jose, CA, USA).

### 4.8 Transfection

Cells were transiently transfected with siRNA using Lipofectamine RNAiMAX (Thermo Fisher Scientific) or with plasmids using Lipofector p‐MAX (AptaBio, Yongin, South Korea).

### 4.9 Quantitative PCR

Total RNA isolation, complementary DNA synthesis, and qPCR analysis were performed as previously described [38]. The amplification of mRNA was assessed using a Thermal Cycler Dice Real‐Time System III (Takara Bio Inc., Shiga, Japan) and determined using the 2^−ΔΔCt^ method. The primers used for gene amplification are listed in Table S3 (Supporting Information).

### 4.10 Biotin Pull‐Down Assay

Cell were lysed in a CHAPS lysis buffer containing protease cocktail (Roche, Mannheim, Germany). The lysates were incubated with Binder_13_‐biotin for 1 h and subsequently incubated with streptavidin Magnetic Beads (New England Biolabs, Ipswich, MA, USA) overnight at 4°C with rotation to capture biotin‐labeled peptide‐protein complexes. After centrifugation, the supernatants were discarded, and the beads were washed with lysis buffer. The bound proteins were eluted and boiled in were boiled in 2× sample buffer, and interaction between peptide and targets protein were detected by western blotting.

### 4.11 Co‐Immunoprecipitation Assay

Cells were co‐transfected with Myc‐SHP2 and Flag‐OTUB1 plasmids. The cells lysed by CHAPS lysis buffer were incubated with 100 µM R7IO‐pep for 1 h and subsequently incubated with the indicated primary antibodies overnight. Then, the lysates were mixed with protein G agarose beads (Santa Cruz Biotechnology, Santa Cruz, CA, USA) for 2 h. The supernatants were removed by centrifugation, and the pellets were boiled in 2× sample buffer. The induced proximity between SHP2 and OTUB1 were detected by western blotting.

### 4.12 Ubiquitination Assay

A ubiquitination assay was performed using a tagged ubiquitin plasmid after pretreatment with MG132. Briefly, the cells were harvested, washed with phosphate‐buffered saline (PBS) containing 10 mM *N*‐ethylmaleimide (NEM; Merck Millipore, Darmstadt, Germany), resuspended in 90 µL PBS/NEM containing 1% SDS, and boiled for 10 min at 95°C. The lysates were added to lysis buffer containing 1 mM phenylmethylsulfonyl fluoride and 5 mM NEM, resuspended, and centrifuged at 13,000 × *g* for 10 min at 4°C. The supernatant was incubated overnight with primary antibodies against the target proteins and then incubated with protein G agarose beads. After centrifugation, the supernatant was removed, washed twice with lysis buffer, and boiled in 2× sample buffer for 10 min. Ubiquitinated target proteins were detected using horseradish peroxidase‐conjugated anti‐Ub antibodies.

### 4.13 Mitochondrial Dysfunction

To detect the source of ROS production, cells were transfected with pHyPer‐cyto, pHyPer‐nuc, or pHyPer‐dMito plasmids (Evrogen, Moscow, Russia). Mitochondrial ROS generation was analyzed using 2 µM MitoSOX Red mitochondrial superoxide indicator (Thermo Fisher Scientific). ROS production was measured using a BD Accuri C6 cytometer (BD Biosciences). To analyze mitochondrial dynamics, mitochondrial morphology in Caki‐1/pDsRed2‐Mito cells was observed using a fluorescence microscope (Carl Zeiss, Jena, Germany). Mitochondrial length (>10 µm was considered elongated and <10 µm as fragmented) was quantified using ZEN3.4. The average length of at least 20 mitochondria was measured for each dataset in three independent experiments.

### 4.14 In Vivo Xenograft Model

Female BALB/c‐nude mice were purchased from JA BIO, Inc. (Suwon, South Korea) and maintained in a pathogen‐free conditioned room. The IRB Keimyung University Ethics Committee (KM‐2020‐03R2) approved the study protocol. MDA‐MB231‐Luc cells were subcutaneously injected on each flank of mice. After three weeks, mice were randomly divided into three groups and intravenously injected with 10 mg/kg bioPhosTAC three times a week. Tumor size (length × width^2^)/2 was measured using a Vernier's caliper (Mytutoyo Co., Tokyo, Japan). The fluorescent signals remaining in major organs and tumors were measured using an IVIS imaging system. Apoptosis was detected with an ApopTag Fluorescein in situ Apoptosis Detection Kit (Millipore), according to the manufacturer's instructions.

### 4.15 Statistical Analysis

All statistical analyses were performed using the Statistical Package for the Social Sciences (SPSS, v.26.0; IBM Corp., Armonk, NY, USA), with statistical significance set at p < 0.05. All experiments were conducted in triplicate. Data are presented as mean ± standard deviation (SD) and were analyzed using one‐way analysis of variance, followed by post hoc comparisons (Student–Newman–Keuls test).

## Author Contributions

**Soeun Rhee**: investigation. **Donghyuk Shin**: writing – review and editing, writing – original draft, investigation, funding acquisition. **Seung Un Seo**: investigation, validation. **Taeg Kyu Kwon**: conceptualization, writing – original draft, writing – review and editing, funding acquisition, supervision, project administration. **Minhyeong Choi**: investigation. **Kwang‐Su Park**: conceptualization, writing – original draft, writing – review and editing, methodology. **Seon Min Woo**: validation, investigation, writing – review and editing, visualization, formal analysis. **Simmyung Yook**: formal analysis.

## Funding

This work was supported by the National Research Foundation of Korea (NRF) grant funded by the Korean Government (MSIT and MOE) (Nos. RS‐2024‐00406114, RS‐2024‐00348057, 2021R1C1C100396112, and RS‐2025‐18362970). This research was supported by the Bio&Medical Technology Development Program of the National Research Foundation (NRF) funded by the Korean government (MSIT) (No. RS‐2022‐NR067425). This research was supported by the National Institute of Health (NIH) research project (Nos. 2025‐ER2011‐00, 2025‐ER2011‐01).

## Conflicts of Interest

The authors declare no conflicts of interest.

## Supporting information




**Supporting File**: advs77265‐sup‐0001‐SuppMat.docx.

## Data Availability

The data that support the findings of this study are available from the corresponding author upon reasonable request.
