# Supplementary material for: Selective Modulation of OTUB1 Noncanonical Function via a bioPhosTAC Strategy
Source: Adv Sci (Weinh). 2026 Aug 20;13(62):e77265. doi: 10.1002/advs.77265 (PMC13491355; doi:10.1002/advs.77265)
Supplement: Supplementary file 1 [file ADVS-13-e77265-s001.docx]

Supporting Information

**Selective Modulation of OTUB1 Noncanonical Function via a bioPhosTAC Strategy**

*Seung Un Seo, Seon Min Woo, Minhyeong Choi, Soeun Rhee, Simmyung Yook, Donghyuk Shin^*^, Kwang-Su Park^*^, Taeg Kyu Kwon^*^*

*Corresponding Author

**Figure S1.** Structural context of OTUB1 phosphorylation sites and characterization of recombinant proteins. A) Predicted three-dimensional structure of OTUB1 showing the distinct spatial positions of Y26 and S16. B) SDS–PAGE analysis of purified recombinant OTUB1 and SHP2 proteins.

**Figure S2.** Additional mechanistic characterization of bioPhosTAC. A) Western blotting of protein expression in MDA-MB231 cells treated with 5–10 μM KOI-peptide for 24 h compared with overexpression of OTUB1 wild type (WT) and the catalytically inactive C91S mutant. B) Western blotting of protein expression in MDA-MB231 cells treated with 2.5–10 μM KOI-WT-pep for 24 h. C) Control sensorgrams for the ternary-complex BLI assay shown in Figure 3D. Representative BLI sensorgrams obtained using 20 μM OTUB1 and 50 μM KOI-pep are shown together with the corresponding OTUB1-alone, KOI-pep-alone, and nonspecific-binding controls (sensor only). D) Immunoprecipitation analysis of the OTUB1-E2 enzymes interaction in MDA-MB231 cells treated with KOI-WT-pep and KOI-EA-pep for 24 h.

**Figure S3.** Validation of KKLC-1 expression and tumor-targeting properties of KOI-pep. A, B) Flow cytometry analysis (A) and subcellular fractionation analysis (B) of the surface-associated KKLC-1 expression in MDA-MB231 cells transfected with control or KKLC-1 siRNA. CD44 and DR5 were used as plasma membrane markers. C) Representative *in vivo* bioluminescence (left) and fluorescence (right) imaging of MDA-MB231-Luc xenograft established in BALB/c-nude mice after intravenous injection of 10 mg/kg KOI-WT-Cy5-pep. *p <0.05 compared with control siRNA.

**Figure S4.** KOI-OTU Mut-pep abolishes target affinity and downstream signaling. A) Schematic view of the designed bioPhosTAC according to SHP2 mutation. B) Binding kinetics measurements of OTUB1-Binder_13_ (Q2A, R5A, R13A) to OTUB1_40–271_ were determined using BLI. One representative experiment is shown; similar results were obtained in independent experiments. C) Flow cytometry analysis of the sub-G1 population and western blotting of protein expression in MDA-MB231 cells treated with 2.5–10 μM KOI-WT-pep or KOI-OTU Mut-pep treatment for 24 h. *p <0.05 compared with the control.

**Figure S5.** SHP2 or KKLC-1 contribute to KOI-pep-mediated mitochondrial dysfunction. A–C) Flow cytometry analysis of mitochondrial ROS levels (A), fluorescence micrographs of mitochondrial length (B), and western blotting of protein expression (C) in Caki cells pretreated with 10 μM SHP099 followed by 5–10 μM KOI-pep treatment for 9 h (A,B) or 24 h (C). D–F) Flow cytometry analysis of mitochondrial ROS levels (D), fluorescence micrographs of mitochondrial length (E), and western blotting of protein expression (F) in Caki cells transfected with control or SHP2 siRNA followed by 5–10 μM KOI-pep treatment for 9 h (D,E) or 24 h (F). G–I) Flow cytometry analysis of mitochondrial ROS levels (G), fluorescence micrographs of mitochondrial length (H), and western blotting of protein expression (I) in Caki cells transfected with control or KKLC-1 siRNA followed by 5–10 μM KOI-pep treatment for 9 h (G,H) or 24 h (I). J–L) Flow cytometry analysis of mitochondrial ROS levels (J), fluorescence micrographs of mitochondrial length (K), and western blotting of protein expression (L) in Caki cells pretreated with 10 μM CPZ or 5 μM filipin followed by 5–10 μM KOI-pep treatment for 9 h (J,K) or 24 h (L). Values shown in the graphs represent the mean ± SD of results from three independent experiments. *p <0.05 compared with KOI-WT-pep.

**Figure S6.** Analysis of HPLC and mass spectrometry to verify the purity and identity of the peptides.

**Supplementary Table S1.** 9 selected peptide candidate sequences and their corresponding confidence metrics

| Design | Sequence | pae_interaction | plddt_total |
| --- | --- | --- | --- |
| 1 | SQAAQDAVIAANRAYIASR | 5.297 | 95.255 |
| 2 | SQEEQDKVIEENRKLIESR | 5.302 | 95.446 |
| 3 | SQEEQDKVIEENRKYIKSR | 5.486 | 95.374 |
| 4 | DQAAQDAVIAANRAYIASR | 5.669 | 95.134 |
| 5 | DQAARDAVIAANRAYIKSR | 5.725 | 95.185 |
| 6 | SQEERDKVIAENRAYIKSR | 5.822 | 95.226 |
| 7 | SQAAQDAVLAENRALIASR | 5.841 | 95.105 |
| 8 | SQEAQDEVIAKNREYIKSR | 5.859 | 95.193 |
| 9 | SQEEQDKVIAANREYIKSR | 5.950 | 95.181 |

**Supplementary Table S2.** List of antibodies.

| Antibody | Source | Cat # |
| --- | --- | --- |
| β-actin | Sigma-Aldrich | A2228 |
| Phopsho-OTUB1 (S16) | Affinity Biosciences | AF3558 |
| OTUB1 | Santa Cruz Biotechnology | sc-130458 |
| Raptor | Cell Signaling Technology | 2280S |
| SHP2 | Cell Signaling Technology | 3752S |
| Snail | Cell Signaling Technology | 3879S |
| FoxM1 | Abcam | ab207298-1 |
| cIAP1 | Abcam | ab154525 |
| p53 | Santa Cruz Biotechnology | sc-126 |
| Deptor | Cell Signaling Technology | 11816S |
| MDMX | Santa Cruz Biotechnology | sc-74407 |
| Phospho-EGFR (Y1068) | Cell Signaling Technology | 3777S |
| EGFR | Cell Signaling Technology | 4267S |
| Phospho-STAT1 (Y701) | Santa Cruz Biotechnology | sc-136229 |
| STAT1 | Cell Signaling Technology | 9172S |
| Phospho-ERK (T202/Y204) | Cell Signaling Technology | 9101S |
| ERK | Cell Signaling Technology | 9102S |
| PARP | Cell Signaling Technology | 9542S |
| Phospho-Src (Y416) | Cell Signaling Technology | 2101S |
| Src | Merck Millipore | 05-184 |
| Phospho-JAK2 (Y1067/1068) | Cell Signaling Technology | 3771S |
| JAK2 | Santa Cruz Biotechnology | sc-294 |
| p-STAT3 (Y705) | Santa Cruz Biotechnology | sc-8001 |
| STAT3 | Santa Cruz Biotechnology | sc-8019 |
| KKLC-1 | Abcam | ab219971 |
| Ub-HRP | Enzo Life Sciences | BML-PW0150 |
| Ub | Santa Cruz Biotechnology | SC-8017 |
| Total OXPHOS | Abcam | ab110413 |
| MnSOD | Sigma-Aldrich | 06-984 |
| Mfn1 | Santa Cruz Biotechnology | sc-166644 |
| Mfn2 | Santa Cruz Biotechnology | sc-100560 |
| OPA1 | BD Biosciences | 612606 |
| p-DRP1 (S616) | Cell Signaling Technology | 3455S |
| DRP1 | Cell Signaling Technology | 8570S |
| Fis1 | Santa Cruz Biotechnology | sc-376447 |
| Bim | EMD Millipore | AB17003 |
| Caspase-3 | Enzo Life Sciences | ADI-AAP-113 |

**Supplementary Table 3.** List of primer sequences.

| Genes | Forward | Reverse |
| --- | --- | --- |
| β-actin | TGGGGTGTTGAAGGTCTC | CTACAATGAGCTGCGTGTG |
| Bim | TTCAGCCTGCCTCATGGAA | CACAAAACCCCAAGTCCTCCTT |
